# Comparative Study of the Two Methods of Cervical Length Measurement in the First Trimester for the Prediction of Spontaneous Preterm Labor

**DOI:** 10.7759/cureus.112726

**Published:** 2026-07-15

**Authors:** Shruti Vkp, Sunil Kumar Samal, Udhayalakshmi Thirumal, Suriya Anand, Arivarasan Barathi

**Affiliations:** 1 Obstetrics and Gynaecology, Mahatma Gandhi Medical College and Research Institute, Puducherry, IND; 2 Community Medicine, Employees' State Insurance Corporation (ESIC) Medical College and Hospital, Chennai, IND

**Keywords:** cervical length, diagnostic accuracy analysis, preterm birth, receiver operating characteristic (roc) curve analysis, transvaginal ultrasonography (tvs)

## Abstract

Background: Spontaneous preterm birth remains a leading cause of neonatal morbidity and mortality worldwide. Early identification of women at risk enables timely preventive interventions. This study compared the predictive performance of one-line and two-line first-trimester transvaginal cervical length measurement methods for predicting spontaneous preterm birth.

Methods: This prospective cohort study enrolled 100 women with singleton pregnancies who attended a tertiary care hospital in Puducherry for routine first-trimester nuchal translucency screening between July 2024 and December 2025. During the 11-13+6 weeks' gestational window, transvaginal ultrasonography was used to assess cervical length using both the one-line and two-line measurement techniques. Participants were subsequently followed through pregnancy until delivery, with spontaneous preterm birth before 37 completed weeks considered the primary study outcome. The predictive performance of the two cervical length measurement methods was evaluated and compared using receiver operating characteristic (ROC) curve analysis.

Results: The mean maternal age was 26.9±3.37 years. Most participants were aged 25-30 years (69, 69%) and multiparous (61, 61%). Spontaneous preterm birth occurred in 19 women (19%), while 81 (81%) delivered at term. Cervical length progressively decreased with earlier gestational age at delivery using both the single-line (36.3±4.22 mm, 31.4±3.35 mm, and 29.2±1.70 mm for term, 32-36+6 weeks, and <32 weeks, respectively; p<0.001) and two-line methods (39.7±4.05 mm, 34.3±3.16 mm, and 32.5±3.46 mm, respectively; p<0.001). The mean cervical length was significantly greater with the two-line method than the single-line method (38.6±4.46 mm vs. 35.3±4.52 mm; p<0.001). ROC curve analysis demonstrated superior predictive performance of the two-line method (AUC=0.860; sensitivity 82.72%; specificity 89.47%; cut-off 37 mm) compared with the single-line method (AUC=0.840; sensitivity 81.48%; specificity 89.47%; cut-off 33.7 mm).

Conclusions: Assessment of cervical length in early pregnancy is a reliable tool for predicting the risk of spontaneous preterm birth. Although both methods demonstrated good diagnostic accuracy, the two-line method showed marginally superior predictive performance and may be the preferred technique for early risk stratification and targeted preventive interventions.

## Introduction

Preterm birth is a major challenge to modern obstetrics and a leading cause of morbidity and mortality in the newborn population worldwide. According to the World Health Organization (WHO), preterm birth refers to the delivery of a live infant before the completion of 37 weeks of gestation. Based on gestational age at birth, preterm deliveries are further classified into three categories: extreme preterm (<28 weeks), very preterm (28-31 weeks), and moderate to late preterm (32-36 weeks). Preterm birth continues to contribute a substantial morbidity and mortality burden to the newborn population worldwide, despite advances in the care of the preterm infant and the availability of high-tech facilities for intensive care of the newborn [1]. 

Every year, 15 million preterm births occur worldwide, which account for about 10-11% of all births. Developing countries like India contribute a high percentage of total preterm births worldwide. The country has a high proportion of preterm births in the global prevalence figures. The country also has nearly a quarter of all preterm births globally [2]. Preterm births represent about 35% of all deaths within the newborn population. A large proportion of deaths among children under the age of five are caused by babies being born too early [3].

Spontaneous preterm birth is multifactorial and results from complex interactions between many determinants. A few of the important risk factors are previous preterm birth, cervical insufficiency, uterine anomalies, infection, multiple gestations, and inflammatory processes. However, the process has become more challenging due to a considerable number of cases of spontaneous preterm birth without any associated risk factors [4].

Recently, there has been great interest in the identification of early predicting factors of spontaneous preterm birth. The length of the uterine cervix is one of the most important anatomical predicting factors of spontaneous preterm birth. Shortening of the uterine cervix during pregnancy is associated with structural and biochemical changes of the cervical tissues prior to the onset of labor. This may be viewed as an early predictor of spontaneous preterm birth [5].

Ultrasonographic measurement of cervical length has been shown to be an important method for assessing the risk of spontaneous preterm birth. Transvaginal ultrasonography performed during the second trimester has long been considered the reference standard for cervical length assessment because of its excellent accuracy, reliability, and reproducibility. However, the ability to identify women at risk of preterm birth in the second trimester may limit the opportunity to provide early intervention [6].

With the growing popularity of first-trimester screening programs, the importance of measuring cervical length in early pregnancy is also increasing. Cervical length measurement in the first trimester could provide a potential method of identifying those women who are likely to go into spontaneous preterm labor in pregnancy. Early diagnosis will enable obstetricians to intervene in a timely manner to prevent preterm labor and improve pregnancy outcomes significantly [7].

There are different ultrasonographic methods to assess cervical length during pregnancy: transperineal, transvaginal, and transabdominal ultrasonography. Transvaginal ultrasonography is the preferred modality for measuring cervical length during pregnancy because it provides a clear view of the internal and external os during pregnancy without interference from maternal obesity and bladder fullness. Nuchal translucency (NT) measurement during routine first-trimester screening is often combined with transabdominal ultrasonography. If this technique is proven to be reliable for measuring cervical length during pregnancy, it may provide a potential screening tool without requiring any additional procedure during pregnancy [8].

Researchers have tried to evaluate the different cervical length measurement techniques for their reliability in predicting spontaneous preterm birth in pregnancy. Measurement of cervical length in the second trimester is widely used in the prediction of preterm birth during pregnancy, but fewer studies have assessed measurement of cervical length in the first trimester of pregnancy. There is a paucity of evidence on different methods of cervical length measurement during early pregnancy [9].

The assessment of the efficacy of methods of cervical length during the first trimester is relevant from a clinical point of view considering the significant problem of preterm birth in India and the need to adopt efficient methods of early prediction of the occurrence of the problem. A comparison of the efficacy of two methods of cervical length measurement may provide valuable insight into the method which is more accurate in predicting the onset of spontaneous preterm labor. Against this background, the present study was undertaken to evaluate and compare the effectiveness of the single-line and two-line methods of first-trimester cervical length measurement in predicting spontaneous preterm birth.

## Materials and methods

Study design and setting

This hospital-based prospective cohort study was conducted among pregnant patients attending the outpatient department of Mahatma Gandhi Medical College and Research Institute, a tertiary care hospital in Puducherry, India, for their first-trimester NT scan. The study was carried out in the Obstetrics and Gynaecology outpatient department for 18 months, from July 2024 to December 2025. The institution is a tertiary care teaching hospital catering to a diverse population from urban and rural areas, providing comprehensive obstetric care including antenatal screening, delivery services, and neonatal care. The study was designed to prospectively evaluate and compare the predictive accuracy of two distinct methods of cervical length measurement, the single-line method and the two-line method, performed during the first-trimester NT scan, for predicting spontaneous preterm birth. This prospective design was chosen to ensure standardized data collection, minimize recall bias, and allow systematic follow-up of all participants until delivery.

Study population and sampling

The study population comprised all pregnant patients attending the outpatient department for their first-trimester NT scan who planned to deliver at the same institution. This ensured complete follow-up and availability of perinatal outcome data. A consecutive sampling technique was employed, wherein all eligible women presenting during the study period who fulfilled the inclusion criteria and provided written informed consent were recruited until the required sample size was achieved. This approach minimized selection bias and ensured representativeness of the clinical population attending the antenatal clinic.

Inclusion and exclusion criteria

All pregnant patients attending the outpatient department for their NT scan and planning to deliver at the tertiary care hospital were included in the study. Patients below 18 years of age were excluded to avoid inclusion of adolescent pregnancies with different risk profiles. Multifetal gestations were excluded due to the higher baseline risk of preterm birth and different pathophysiology compared to singleton pregnancies. Patients with fetal anomalies detected on the NT scan were excluded as these may independently influence pregnancy outcomes and cervical remodeling. Women with previous cervical surgeries or pathology, including cervical conization, loop electrosurgical excision procedure (LEEP), or cervical cerclage, were excluded as these interventions alter cervical architecture and may affect cervical length measurements. Patients with a history of pelvic irradiation were excluded due to potential radiation-induced cervical changes. Women who declined to participate or did not provide written informed consent were excluded from the study.

Sample size determination

The sample size was calculated based on the study conducted by Feng et al. [10], where the cervical length measured by the single-line method was reported as 33.5±16.7 mm and by the two-line method as 36.5±16.7 mm from previous research. The sample size was estimated using the formula n=(Z²×σ²)/d², where Z represents the standard normal deviate corresponding to a 95% confidence level (1.96), σ² is the variance (278.89), and d is the precision set at 10% of the mean (3.35). Applying the formula, n=(3.84×278.89)/11.22, which yielded 95.4 participants. The final sample size was rounded to 100 participants to account for potential dropouts and incomplete data, ensuring adequate statistical power to detect clinically meaningful differences between the two measurement methods.

Study procedure

After obtaining approval from the Institutional Human Ethics Committee of Mahatma Gandhi Medical College and Research Institute (approval number: MGMCRI/Res/01/2023/76/IHEC/38), eligible participants were approached during their routine NT scan. All eligible participants received a comprehensive explanation of the study objectives, procedures, potential risks, and anticipated benefits. Written informed consent was obtained from each woman prior to enrolment in the study. Baseline demographic and clinical characteristics were collected using a structured proforma, including maternal age, gestational age, parity, body mass index (BMI), obstetric history, medical comorbidities, and any previous history of preterm birth.

Following enrollment, all participants underwent a standard first-trimester NT scan between 11 and 14 weeks of gestation using a GE Voluson P8 ultrasound machine (GE Healthcare, Chicago, Illinois, United States) equipped with a high-frequency vaginal transducer (3-12 MHz). The NT scan was performed by a single experienced sonographer to ensure consistency and eliminate inter-observer variability in cervical length measurements. Prior to the procedure, participants were instructed to empty their bladder to avoid artifactual elongation of the cervix. The patient was positioned in a semi-recumbent position with abducted legs. The vaginal transducer was lubricated with gel on both sides, taking care to remove air bubbles to optimize acoustic coupling. The transvaginal probe was carefully inserted into the anterior vaginal fornix and manipulated to obtain a clear mid-sagittal image encompassing the full length of the endocervical canal. Care was taken to avoid exerting undue pressure on the cervix, ensuring that both cervical lips had the same width to prevent artifactual shortening or lengthening of the cervical canal. The ultrasound image was enlarged until the cervix occupied approximately 50-75% of the display, ensuring optimal visualization and accurate cervical length measurement.

Cervical length was measured by two distinct methods in the same participant. For the one-line technique, cervical length was determined by drawing a straight line along the endocervical canal, extending from the internal os to the external os, with the measurement recorded in millimeters. In the two-line method, two lines were drawn, one line from the internal os along the anterior cervical wall and a second line from the internal os along the posterior cervical wall, with the cervical length measured as the mean distance between the two lines. Both measurements were recorded for each participant. Additional findings were documented during the examination, including the presence of funneling, amniotic fluid debris, sludge, membrane separation, vasa previa, low-lying placenta, or abnormally invasive placenta. To ensure accurate cervical length assessment, potential sources of measurement error were minimized by avoiding bladder overdistension and excessive transducer pressure, both of which can falsely increase cervical length. Care was also taken to distinguish true cervical funneling from transient uterine contractions or thickening of the lower uterine segment.

Follow-up and outcome assessment

All enrolled women were prospectively followed from recruitment until delivery at the hospital. The primary study outcome was spontaneous preterm birth, defined as delivery before 37 completed weeks of gestation (<259 days) following spontaneous onset of labor or preterm prelabor rupture of membranes. Women who underwent planned induction of labor or elective cesarean delivery before 37 weeks for maternal or fetal indications were not considered to have experienced spontaneous preterm birth. Perinatal outcomes, including gestational age at delivery, mode of delivery, birth weight, and neonatal complications, were obtained from hospital delivery records.

Data analysis

Data collected using a structured case record form were entered into Microsoft Excel (Microsoft Corporation, Redmond, Washington, United States) and analyzed using IBM SPSS Statistics for Windows, Version 25.0 (IBM Corp., Armonk, New York, United States). Continuous variables, including maternal age, gestational age, BMI, and cervical length measured by the single-line and two-line techniques, were summarized as mean±standard deviation (SD) for normally distributed data or as median with interquartile range (IQR) for non-normally distributed data. Categorical variables, such as parity, previous history of preterm birth, and spontaneous preterm birth, were expressed as frequencies and percentages.

The normality of continuous variables was assessed using the Shapiro-Wilk test. Cervical length measurements obtained using the single-line and two-line methods in the same participants were compared using the paired t-test for normally distributed data or the Wilcoxon signed-rank test when the normality assumption was not met. Differences in cervical length across gestational age categories at delivery were evaluated using one-way analysis of variance (ANOVA) for normally distributed data or the Kruskal-Wallis test for non-parametric data.

Receiver operating characteristic (ROC) curve analysis was performed to evaluate the predictive performance of both cervical length measurement techniques for spontaneous preterm birth. The area under the ROC curve (AUC), sensitivity, specificity, positive predictive value (PPV), and negative predictive value (NPV) were calculated for each method. The optimal threshold for predicting spontaneous preterm birth was identified using the Youden index. The diagnostic performance of the single-line and two-line methods was compared by evaluating their respective AUCs. A two-sided p-value of <0.05 was considered statistically significant for all analyses.

## Results

The baseline demographics, obstetric characteristics, cervical length measurements, and pregnancy outcomes of the study participants are summarized in Table 1. The mean age was 26.9±3.37 years, with the majority belonging to the 25-30-year age group (69, 69%), followed by those aged 18-24 years (18, 18%) and >30 years (13, 13%). Most women were multiparous (61, 61%), while 39 (39%) were primiparous. Cervical length was most commonly measured between 12 and 12+6 weeks of gestation (56, 56%), followed by 13-13+6 weeks (30, 30%) and 11-11+6 weeks (14, 14%). A previous history of preterm delivery was present in 20 (20%) participants, whereas seven (7%) had undergone previous cervical surgery. Most pregnancies resulted in term delivery (81, 81%), while 19 (19%) delivered preterm, corresponding to a preterm birth rate of 19% (95% CI: 11.8-28.1%). 

**Table 1:** Baseline demographics, obstetric characteristics, and pregnancy outcomes of the study participants (N=100)

Variable	Category	n	%
Age group (years)	18-24	18	18
	25-30	69	69
	>30	13	13
Parity	Primiparous	39	39
	Multiparous	61	61
Gestational age at cervical length measurement	11-11+6 weeks	14	14
	12-12+6 weeks	56	56
	13-13+6 weeks	30	30
Previous preterm delivery	Yes	20	20
	No	80	80
Previous cervical surgery	Yes	7	7
	No	93	93
Gestational age at delivery	<32 weeks	2	2
	32-36+6 weeks	17	17
	≥37 weeks	81	81
Pregnancy outcome	Preterm delivery (<37 weeks)	19	19
	Term delivery (≥37 weeks)	81	81

Table 2 compares the mean cervical length measured by the single-line and two-line methods across gestational age groups at delivery. Women who delivered at term (>37 weeks) had the highest mean cervical length using both the single-line method (36.3±4.22 mm) and the two-line method (39.7±4.05 mm). The mean cervical length progressively decreased among women delivering between 32 and 36+6 weeks (31.4±3.35 mm and 34.3±3.16 mm, respectively) and was lowest among those delivering before 32 weeks (29.2±1.70 mm and 32.5±3.46 mm, respectively). One-way ANOVA demonstrated a statistically significant difference in cervical length across the three gestational age groups for both measurement methods (p<0.001). Bonferroni post hoc analysis further confirmed differences between all pairwise gestational age groups, indicating that cervical length decreased significantly with earlier gestational age at delivery irrespective of the measurement technique.

**Table 2:** Comparison of cervical length measurements according to gestational age at delivery using the single-line and two-line methods One-way analysis of variance followed by Bonferroni post hoc test; statistically significant at p<0.05. Bonferroni post hoc analysis demonstrated statistically significant differences between all pairwise gestational age groups (>37 vs. 32-36+6 weeks, >37 vs. <32 weeks, and 32-36+6 vs. <32 weeks) for both the single-line and two-line cervical length measurement methods. *p-value <0.05 is statistically significant

Method	>37 weeks (mean±SD)	32-36+6 weeks (mean±SD)	<32 weeks (mean±SD)	F value	P-value
Single-line method (mm)	36.3±4.22	31.4±3.35	29.2±1.70	41.7	<0.001*
Two-line method (mm)	39.7±4.05	34.3±3.16	32.5±3.46	36.6	<0.001*

The mean cervical length measured using the two-line method (38.6±4.46 mm) was significantly greater than that measured using the single-line method (35.3±4.52 mm) (p<0.001), indicating a statistically significant difference between the two measurement techniques.

**Figure 1:**
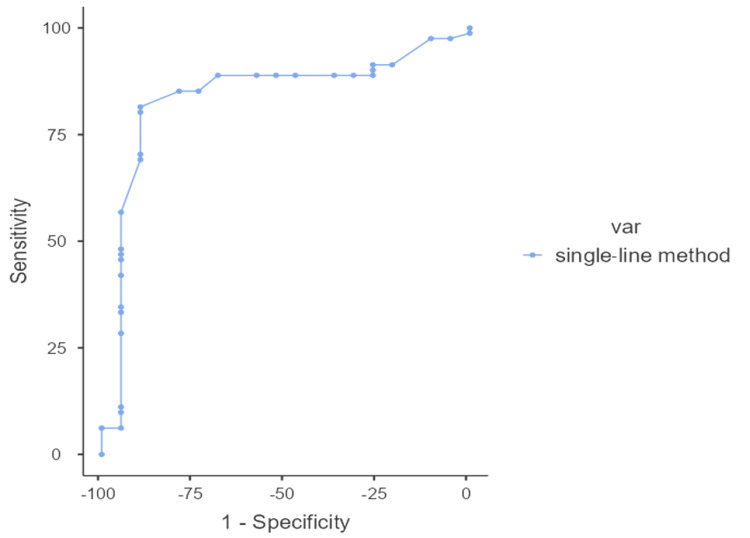
Receiver operating characteristic curve of the single-line method to predict preterm delivery

As shown in Figure 1, the ROC curve analysis of the single-line method of cervical length measurement was done to predict preterm delivery in our study participants. The cutoff value of cervical length in mm by the single-line method to predict preterm delivery was 33.7 with a maximum AUC of 0.840. 

**Figure 2:**
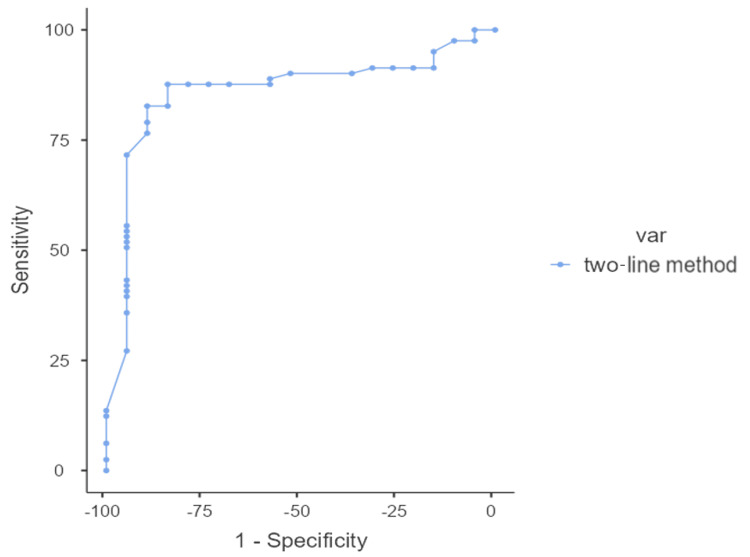
Receiver operating characteristic curve of the two-line method to predict preterm delivery

As shown in Figure 2, the ROC curve analysis of the two-line method of cervical length measurement was done to predict preterm delivery in our study participants. The cutoff value of cervical length in mm by the two-line method to predict preterm delivery was 37 with a maximum AUC of 0.860.

The predictive values of the single-line and two-line methods to predict preterm delivery were compared in Table 3. The two-line method had a higher AUC (0.860 vs. 0.840) and slightly better sensitivity (82.72% vs. 81.48%) which means that it was better at making predictions. The specificity of both methods was equal (89.47%). The PPVs for both methods were very high (>97%) meaning that both methods were very good in detecting preterm birth when the cervical length was below the cut-off. However, their lower NPVs suggest they may not be very good at ruling out preterm delivery. The two-line method appears to be a better and clinically more reliable method of predicting preterm delivery in early pregnancy.

**Table 3:** Comparison of predictive values of the single-line and two-line methods

Parameter	Single-line method	95% CI	Two-line method	95% CI
Cut-off (mm)	33.7	-	37.0	-
Sensitivity (%)	81.48	71.67-88.44	82.72	73.05-89.42
Specificity (%)	89.47	68.61-97.06	89.47	68.61-97.06
Positive predictive value (%)	97.06	89.90-99.19	97.10	90.03-99.20
Negative predictive value (%)	53.12	36.45-69.13	54.84	37.77-70.84
Area under the curve	0.840	0.758-0.922	0.860	0.784-0.936

## Discussion

The present prospective cohort study was conducted to evaluate and compare the predictive performance of the single-line and two-line methods of first-trimester cervical length measurement for identifying women at risk of spontaneous preterm birth. Most participants were in the 25-30-year age group, which is the optimum reproductive age range. Maternal age has been shown to impact pregnancy outcomes, with advanced maternal age associated with increased risk of pregnancy complications including preterm birth [11]. With respect to parity, multiparous women formed the larger proportion of the study population, which is comparable to observations reported in many hospital-based obstetric studies carried out in India. A previous history of preterm birth was seen in a substantial proportion of participants. This finding is clinically significant because a previous spontaneous preterm delivery is one of the strongest predictors of a subsequent preterm birth [12]. Women with a previous history of spontaneous preterm delivery have substantially higher recurrence rates than women without such a history [13]. The inclusion of these women in the present study reflects real-world antenatal practice and increases the clinical applicability of the findings.

Cervical length was measured between 11 and 13 weeks plus six days of gestation during routine NT scanning. The rationale for first-trimester cervical length assessment is based on the increasing incorporation of cervical assessment into routine first-trimester fetal screening programs [14]. At this stage, a measurement allows risk assessment much earlier than the conventional second-trimester cervical screening and may permit earlier preventive interventions. Previous studies have demonstrated that first-trimester cervical assessment at 11-13 weeks could identify women at increased risk of spontaneous preterm birth [7]. Similarly, researchers have found that first-trimester cervical length measurements were useful for prediction models of early preterm delivery [15]. 

The overall incidence of preterm birth observed in this study was higher than the global average reported by the WHO, which estimates that about 10-11% of all births worldwide are preterm [2]. This observed prevalence is not surprising as the study was conducted in a tertiary care referral institution where higher-risk pregnancies are often managed. Preterm birth rates of 10-13% in the general population have been reported in studies in India [16]. Higher-risk pregnancies have been documented in referral hospitals and tertiary centers, and as such, higher rates have been documented. Therefore, the preterm birth rate found in the current study is indicative of the study setting and underscores the ongoing importance of valid screening tools for the early identification of women at risk.

The analysis revealed that the two-line method resulted in longer cervical length measurements compared to the single-line method and this difference was statistically significant. This difference may be due to the anatomic configuration of the cervical canal. The single-line technique determines cervical length by measuring the straight-line distance between the internal and external os. In contrast, the two-line technique accounts for the physiological curvature of the endocervical canal by measuring the canal in two contiguous segments and summing these measurements to obtain the total cervical length [17]. Thus, the two-line method is thought to provide a more anatomically representative estimate of cervical length, especially in the presence of cervical curvature or angulation. The present study results are very similar to the report by Feng and colleagues, who demonstrated that the median cervical length measured using the single-line technique was significantly shorter than that obtained with the two-line technique [10]. In accordance with our results, the authors concluded that the two-line method provided higher cervical length values and better reflected cervical anatomy. The agreement of the present results with those of Feng and colleagues strongly supports the validity of the observed differences between the two measurement techniques.

The present study found a significant association between cervical length and gestational age at delivery, demonstrating a clear inverse relationship between cervical length and risk of preterm delivery. Women with a shorter cervical length in the first trimester had a significantly shorter gestational age at delivery. The biological underpinnings of this association are well characterized. Cervical shortening is a sign of early cervical remodeling with collagen degradation, increased hydration, and loss of tissue integrity [18,19]. These changes occur before clinical presentation, resulting in early cervical incompetence and spontaneous preterm labor [20]. The findings of the present study are consistent with the landmark study by Iams and colleagues, which demonstrated that shorter cervical length is associated with increased risk of spontaneous preterm delivery, showing a strong inverse relationship between cervical length and risk of preterm birth, with progressively shorter cervices predicting delivery at earlier gestational ages [5]. Similarly, Heath and colleagues found that cervical length at 23 weeks was significantly associated with spontaneous preterm birth, with a gradual increase in risk as cervical length decreased [21]. The present study extends these observations to the first trimester and suggests that cervical shortening may be detectable much earlier than is traditionally appreciated.

Carvalho and colleagues measured cervical length at 11-14 weeks and found that women who subsequently delivered preterm had shorter cervical lengths than those who delivered at term [22]. Differences in the first trimester were small, but subsequent shortening over the course of pregnancy was significantly greater among women who delivered preterm. Greco and colleagues reported significantly shorter endocervical lengths at 11-13 weeks in women who subsequently delivered preterm compared with unaffected pregnancies [7]. Likewise, To and colleagues demonstrated that measuring cervical length at 11-14 weeks significantly improved the prediction models for spontaneous early preterm delivery [15]. The findings of the present study are consistent with these observations, demonstrating that women who experienced spontaneous preterm birth had significantly shorter cervical lengths than those who delivered at term. The present study used a single measurement technique, directly compared two measurement techniques, thus providing additional information regarding optimal methodology for first-trimester cervical assessment.

The present study was carried out to assess the predictive value of first-trimester cervical length measurements for spontaneous preterm birth. ROC curve analysis showed that both methods had good predictive accuracy for identifying women at risk of preterm delivery. Both measurement methods demonstrated good discriminatory ability to distinguish between women who would have a subsequent spontaneous preterm delivery and women who would deliver at term. The high sensitivity found in our study suggests that most women who did deliver preterm were correctly identified as high risk. These results support the hypothesis that cervical shortening is one of the strongest sonographic predictors of spontaneous preterm birth [5]. The ability to identify high-risk pregnancies by cervical assessment in the first trimester is of particular importance as many women who experience spontaneous preterm labor have no identifiable clinical risk factors [23]. The high PPV observed indicates that women with cervical lengths below the identified cut-off had a very high likelihood of subsequent preterm delivery. This finding has important clinical implications, suggesting that targeted preventive interventions may be directed to a well-defined high-risk population.

Iams and colleagues demonstrated that women with a short cervical length had a significantly increased risk of spontaneous preterm delivery, providing evidence that cervical length was one of the most potent predictors and laying the foundation for current cervical screening programs [5]. A strong negative correlation between cervical length and spontaneous premature delivery was also found by Heath and colleagues, who found that the shorter the cervical length, the more likely the women were to have a preterm birth [21]. Women who later delivered preterm had significantly shorter cervical lengths in the first trimester, and adding first-trimester cervical length to prediction models increased the detection of women at risk of early preterm birth [7,15]. The present study confirms the same in an Indian population and also shows that the predicted accuracy is dependent on the measuring procedure.

However, a few limitations must be acknowledged. The study was conducted at a single tertiary care institution; therefore, the findings may not be generalizable to other populations or primary care settings. The sample size of 100 participants is small compared to major worldwide studies evaluating cervical length screening, though it was statistically adequate. The limited number of very early preterm births, with only two women giving birth prior to 32 weeks of gestation, precluded detailed subgroup analysis for extremely preterm deliveries. The absence of multivariable analysis means that potential confounders such as maternal BMI, socioeconomic characteristics, infection status, and obstetric history were not included in a prediction model. Finally, the lack of serial cervical length assessment throughout pregnancy means that the dynamics of cervical shortening could not be evaluated.

The findings of this study have several important clinical implications. First-trimester cervical length measurement using either method may act as a helpful screening tool for spontaneous preterm birth. The two-line method appears to be more accurate for the anatomical assessment of cervical length and may be the method of choice for predicting spontaneous preterm delivery in early pregnancy. The identification of women at increased risk early in pregnancy allows the timely implementation of preventive strategies such as vaginal progesterone therapy, cervical cerclage, or enhanced antenatal surveillance. The high NPV of both methods provides reassurance that women with cervical lengths above the identified cut-offs are at low risk, potentially reducing unnecessary interventions and anxiety. Integrating cervical length measurement into routine first-trimester screening programs could improve early risk stratification without requiring additional visits.

Future research should focus on large, multicenter prospective studies with standardized protocols to validate these findings across diverse populations. Studies evaluating serial cervical length measurements throughout pregnancy would provide valuable information on the trajectory of cervical shortening and its predictive value. Incorporating multivariable prediction models that combine cervical length with maternal demographic factors, clinical history, and biochemical markers may improve risk stratification. Research evaluating the cost-effectiveness of first-trimester cervical screening programs would inform healthcare policy decisions, particularly in resource-limited settings. Finally, interventional studies assessing the impact of early preventive strategies based on first-trimester cervical length measurements on preterm birth rates and perinatal outcomes are urgently needed.

## Conclusions

This study demonstrated a significant correlation between cervical length measured in the first trimester and gestational age at delivery later in pregnancy. Women who delivered preterm had significantly shorter cervical lengths compared to those who delivered at term. The two-line method showed slightly longer measurement and slightly better diagnostic accuracy for spontaneous preterm birth compared with the single-line method. Both techniques demonstrated good sensitivity and specificity, and the two-line method had a higher AUC indicating better overall prediction accuracy. The results suggest that cervical length measurement in the first trimester may be a helpful screening tool to identify women at high risk for spontaneous preterm birth. The two-line method appears to be more accurate for the anatomical assessment of cervical length and may be the method of choice for predicting spontaneous preterm delivery in early pregnancy. The identification of optimal measurement techniques and appropriate cut-off values represents an important step toward implementing effective first-trimester screening programs to reduce the burden of preterm birth and its associated neonatal morbidity and mortality.
